# Integrated ultracompact and broadband wavelength demultiplexer based on multi-component nano-cavities

**DOI:** 10.1038/srep27428

**Published:** 2016-06-06

**Authors:** Cuicui Lu, Yong-Chun Liu, Xiaoyong Hu, Hong Yang, Qihuang Gong

**Affiliations:** 1 Qian Xuesen Laboratory of Space Technology, China Academy of Space Technology, Beijing 100094, People’s Republic of China; 2 State Key Laboratory for Mesoscopic Physics & Department of Physics, Peking University, Beijing 100871, People’s Republic of China; 3 Collaborative Innovation Center of Quantum Matter, Beijing 100871, People’s Republic of China

## Abstract

Integrated nanoscale photonic devices have wide applications ranging from optical interconnects and optical computing to optical communications. Wavelength demultiplexer is an essential on-chip optical component which can separate the incident wavelength into different channels; however, the experimental progress is very limited. Here, using a multi-component nano-cavity design, we realize an ultracompact, broadband and high-contrast wavelength demultiplexer, with 2.3 μm feature size, 200 nm operation bandwidth (from 780 nm to 980 nm) and a contrast ratio up to 13.7 dB. The physical mechanism is based on the strong modulation of the surface plasmon polaritons induced by the multi-component nano-cavities, and it can be generalized to other nanoscale photonic devices. This provides a strategy for constructing on-chip photon routers, and also has applications for chip-integrated optical filter and optical logic gates.

The realization of photonic chip is researchers’ long-sought goals. Nanoscale integrated wavelength demultiplexers, which can separate the incident different wavelength to propagate in different channels^1^,^2^, have great potential applications in the fields of integrated devices and circuits, such as photon router, wavelength filter, and all-optical logic gates^1^,^3^,^4^,^5^,^6^,^7^,^8^,^9^,^10^,^11^,^12^,^13^,^14^. Surface plasmon polariton (SPP) devices and circuits, bridging the gap between integrated photonic and microelectronic technology, are promising candidates to realize on-chip ultracompact planar structures^15^,^16^,^17^. The wavelength demultiplexers based on SPPs almost possess the smallest size. The first approach to realize plasmonic wavelength demultiplexers is based on the Brag reflection through designing different gratings, but the bulky periodic configuration considerably increased their sizes. This greatly limits their applications for integration. The second approach is based on the optical interferences through etching asymmetric nano-slits or grooves on gold films directly^5^,^6^,^8^,^11^,^13^. This approach ensures compact size, however, the operation band is not broad and the contrast ratio is not high.

In 2011, Liu *et al.* experimentally realized a submicron plasmonic dichroic demultiplexer on gold film directly, but the highest splitting intensity ratios were only 3:1 (i.e. 4.8 dB) at short wavelengths and 1:2 (i.e. −3.0 dB) ratio at long wavelengths^5^. Subsequently, more plasmonic wavelength demultiplexers were realized based on optical interferences by directly fabricating nano-slits and grooves on gold film^6^,^8^,^13^. The operation band is mainly limited by the optical interference principle, and the contrast ratio is limited by the weaker modulation on SPPs using gold film merely instead of using gold film coated with high-index dielectric layer^18^,^19^. Recently, Piggott *et al.* proposed a novel method through adopting inverse design and implementation to realize a wavelength demultiplexing grating coupler fabricated in silicon on insulator^1^. The novel grating separated 1300 nm and 1550 nm light into different waveguides. The size of the device size was 8 μm, and the device transmission possesses high efficiency, which is almost transparent for infrared wavelength exceeding 1100-nm^1^. Very recently, Piggott *et al.* realized a wavelength demultiplexer that split 1300 nm and 1500 nm light by adopting an algorithm method^2^. The crosstalk was less than −11 dB, the footprint is 2.8 × 2.8 μm^2^, and the bandwidth is more than 100 nm^2^, which attracted great interests of researchers on the integrated wavelength demultiplexer. It is worth mentioning that a polarization beamsplitter with similar structures is also realized by Shen *et al.* by use of the algorithm method^3^,^14^. However, the experimental progress on the integrated wavelength demultiplexer is very limited, so it is still a great challenge to perform an easy experiment to achieve wavelength demultiplexers with broadband operation, ultracompact size, high contrast ratio, simultaneously.

In this paper, we propose to adopt two asymmetric multi-component SPP nano-cavities on the metal/polymer/air configuration to experimentally realize a nanoscale wavelength demultiplexer on the chip. It is easy to coat a polymer layer on the gold film by using a spin-coating method, and the multi-component cavity is easy to fabricate by directly using focused-ion-beam etching on the polymer. The measured plasmonic wavelength demultiplexer operates from 780 nm to 980 nm, the feature size of its whole lateral dimension is only 2.3 μm, and the measured contrast ratio of the left intensity to the right intensity reaches 13.7 dB at 840 nm and −10.3 dB at 920 nm with a transition wavelength of 890 nm (where the left intensity is equal to the right intensity). In such a planar configuration of the ultracompact device, the coupling of free-space signal lights to surface plasmon polaritons and the on-chip demultiplexer are integrated together, which is very suitable for practical integration applications, such as photon sorter, router, filter and even all-optical logic gates^12^,^18^,^19^,^20^,^21^.

## Results

In order to illustrate the design rule for our plasmonic wavelength demultiplexer device, we first investigated the properties of the structure with only one multi-component nano-cavity, especially the mode coupling for SPP and cavity. The nano-slit acts as a photon-to-SPP converter, and it is widely used in a variety of plasmonic devices^6^,^8^,^11^,^13^,^18^,^20^,^22^,^23^,^24^,^25^,^26^. The coupling efficiency between the free-space laser beam and the nano-slit is about 3% for the Au/PVA/Air configuration according to our calculation by using of finite element method. Owing to the symmetric configuration of the nano-slit, there exist left- and right-propagating SPP modes in the interface between gold film and PVA layer. Considering one nano-cavity, i.e. a nano-groove etched on the PVA layer and the gold film, the SPPs will be modulated by the nano-cavity when the SPPs propagate through the nano-cavity. M. Kuttge *et al.* pointed out that the depth and the width of the nano-cavity had important influences on the propagation state of SPPs^27^. If the SPP mode does not match the cavity mode, it will be reflected by the cavity, and there will no SPPs propagating through the nano-cavity; if the SPP mode matches the cavity mode, it will generate resonances in the cavity, and a large part of SPPs will penetrate the cavity and propagate forward. The plasmonic nano-cavity mode generally has a relatively small quality factor (related to the dissipation rate of photons confined to the cavity), or a large linewidth, due to the strong losses of metal^17^,^27^,^28^,^29^,^30^,^31^, so it is possible to achieve a broad operation band for the wavelength demultiplexer by carefully designing the cavity structures. In other words, the smaller of the quality factor for the cavity, the easier for the resonances, and the larger of the operation band^23^.

**Figure 1:**
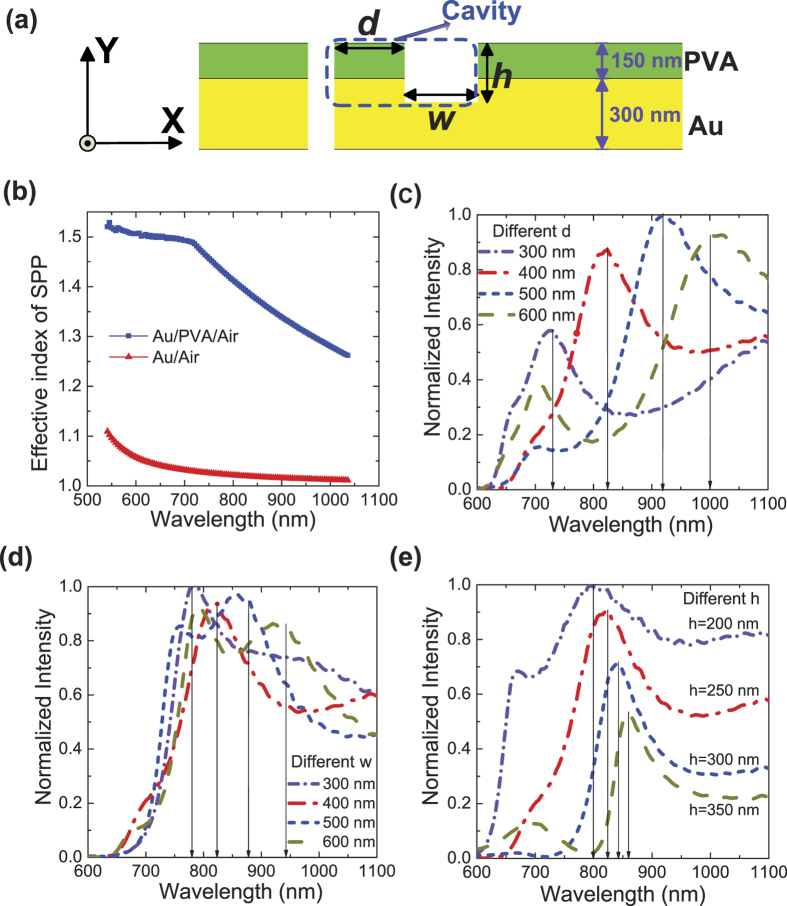
Calculation for the single multi-component cavity (MCC). (**a**) Schematic structure with single MCC. The green layer indicates PVA, the yellow layer indicates gold, and the white area denotes air. “*h*” “*d*” “*w*” are used to denote the key parameters of the MCC. (**b**) Calculated effective index of the SPP mode for the configurations of Au/Air (without polymer layer on) and Au/PVA/Air, respectively. (**c**) Calculated normalized intensity for the right side of the MCC for different *h* (**c**), different *d* (**d**), and different *w* (**e**) of the cavities. The black arrows indicate the corresponding incident wavelengths for the intensity peaks of the same order of SPP mode.

The multi-component nano-cavity (MCC) is composed of three components, including gold, PVA, and air. The cross-section structure of the configuration with only one MCC is shown in Fig. 1(a), where *d* is the distance of the left wall of the air groove to the right wall of the nano-slit, i.e. the length of PVA layer in MCC; *w* is the width of the air groove, i.e. the length of Air layer in MCC; and *h* is the depth of the cavity, i.e. the height of Air layer in MCC. The resonance conditions for the SPPs in the MCC can be approximately described by the formula,


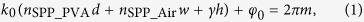


where *k*_0_ is the wave vector for the incident wavelength in the vacuum, which is equal to  2*π*/*λ*; *n*_SPP_PVA_ and *n*_SPP_Air_ are the effective refractive index of the SPP mode in the Au/PVA interface and Au/Air interface, respectively (see Fig. 1b); *γ* is a fitting coefficient according to the numerical simulation results; *φ*_0_ is the sum of phase shifts brought by the reflection from MCC, which is approximate to *π* and not effected by the thickness of gold according to our calculations; and *m* is a positive integer, which denotes the mode order in MCC. The numerical calculations for the effective index of SPP mode were performed by using the finite element method (adopting a commercial software package COMSOL Multiphysics), which is shown in Fig. 1(b). The thickness of gold film and PVA layer are 300 nm and 150 nm, respectively. It is clear that the effective index of SPP on the interface of gold film and PVA layer (*n*_SPP_PVA_) in the Au/PVA/Air configuration is more than that on the interface of gold film and air (*n*_SPP_Air_) in the Au/Air configuration. *n*_SPP_PVA_ is slightly less than 1.5 (the index of PVA), and *n*_SPP_Air_ is slightly greater than 1.0 (the index of Air), due to the unique properties of the evanescent field of SPP. With the increase of the wavelength, *n*_SPP_PVA_ decreases owing to the weaker confinement on SPPs of the 150-nm-thick PVA layer for the longer wavelengths. For simplicity, the used approximate value for *n*_SPP_PVA_ is 1.4 in formula (1), and the used approximate value for *n*_SPP_Air_ is 1.0 (i.e. the average value of the effective index of SPP mode for the Au/Air configuration) in formula (1).

In order to illustrate the role played by the different structure parameters in formula (1), we calculated the intensity at the right side of the MCC, which are shown in Fig. 1(c–e). Considering the influences only brought by the parameter “*d*” on the propagation of SPPs, the calculation is performed by changing *d* and keeping *w* and *h* constant (*w* = 400 nm, *h* = 250 nm), and the result is shown in Fig. 1(c). When *d* increases 100 nm (Δ*d* = 100 nm), the wavelength for the intensity peak increases about 93 nm (Δ*λ* = 93 nm), which means the SPP resonant mode of the MCC increases 93 nm. Perform difference on *d* in formula (1), we can obtain formula (2).


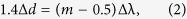


so *m* is equal to 2, which means the second order of SPP mode is applied in our configuration.

Considering the influences only brought by the parameter “*w*” on the propagation of SPPs, the calculation is performed by changing *w* and keeping *d* and *h* constant (*d* = 400 nm, *h* = 250 nm). The result is shown in Fig. 1c. According to formula (1), the influences only brought by the parameter “*w*” can be expressed in formula (3),


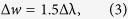


then Δ*λ* = 67 nm, which is agreement with the numerical calculation result in Fig. 1(c).

The influences only brought by the parameter “*h*” on the propagation of SPPs, the calculation is performed by changing *h* and keeping *d* and *w* constant (*d* = 400 nm, *w* = 400 nm). The result is shown in Fig. 1c. We just changed *h* from 200 nm to 350 nm by 50 nm per step, since the total thickness of PVA and Au is limited.


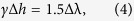


Substituting Δ*h* = 50 nm, Δ*λ* = 23 nm to formula (4), we obtain *γ* is approximate to 0.7. As we know, the SPPs propagate on the interface of Au and PVA, the influences brought by *h* is mainly confined in the vertical direction of the interface, which are different for different configuration, so we adopted a fitting coefficient for evaluating the influences brought by *h*.

It is obvious that *d* has greater impact on the SPPs resonance in the MCC compared with *w*, because the effective index in the Au/PVA/Air configuration is larger than that in the Au/Air configuration; the parameter *h* has the smallest influences on the SPPs resonance of the MCC, since the propagation of SPPs is in the X direction. According to the above theoretical analysis, it is certain to achieve that one MCC takes a high transmission state for the SPPs in a certain frequency range through carefully designing the structural parameters of the two MCCs to satisfy the resonance conditions; at the same time, the other MCC take a reflection state for the SPPs when the SPP mode does not satisfy the resonance conditions. Therefore, it is achievable to design two asymmetric MCCs distributed at the left- and right-side of the nano-slit to separate the incident different wavelength to propagate in two directions. The typical full width at half maximum of the resonance peak for one MCC is more than 150 nm, so it is predicted that the operation band for the wavelength demultiplexer based on two asymmetric MCCs can achieve more than 300 nm.

**Figure 2:**
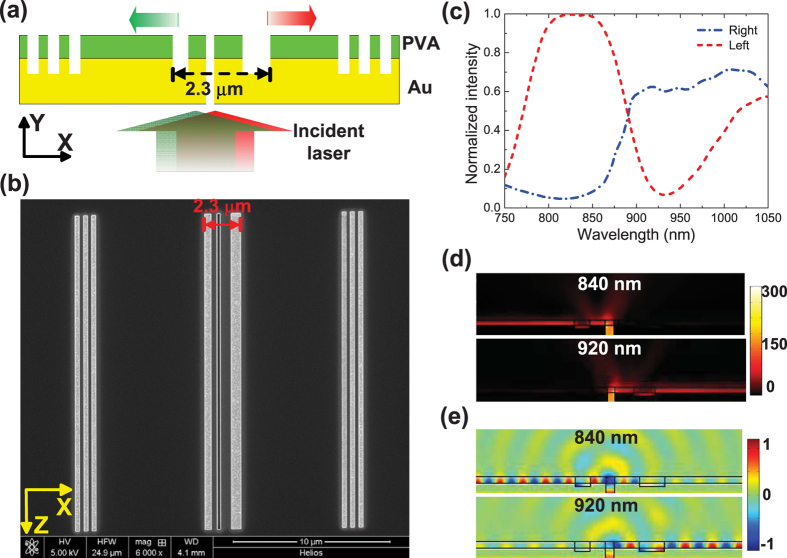
Characteristics of the wavelength demultiplexer. (**a**) Schematic of the demultiplexer structure illuminated by incident laser. The feature size of the device in the X direction is 2.3 μm. The incident laser with short wavelength propagates in the left direction, while the incident laser with long wavelength propagates in the right direction. (**b**) Top-view SEM image of the sample. (**c**) Calculated intensity ratio for the left area of the PVA layer of the left MCC to the right area of PVA layer of the right MCC. Calculated power flow distributions (**d**) and field distributions of Hz component (**e**) on the cross section of the sample for 840 nm and 920 nm, respectively.

The plasmonic wavelength demultiplexer is consisted of a nano-slit and two asymmetric MCCs distributed at the left- and right-side of the nano-slit, respectively. Figure 2(a) shows the schematic of the SPP demultiplexer illuminated by a p-polarized laser (magnetic vector parallel to the slit). Considering the practical measurement using the tunable laser and the etching accuracy, the parameters of the device structure are optimized according to the above equations and the numerical simulations. The width of the air groove at the left side is 400 nm, the width of the air groove at the right side is 650, and the distances of the nano-slit to the left air groove and to the right air groove are 400 nm and 650 nm, respectively, i.e. *d* = *w* = 400 nm for the left MCC, and *d* = *w* = 650 nm for the right MCC. The depth for the nanogrooves is 250 nm, i.e. *h* = 250 nm for the MCCs, which means the PVA layer should be removed completely, and the Au layer should etched 100 nm in depth at the air grooves. The depth and width of the nano-slit were 450 nm and 200 nm, respectively. For the purpose of measurement, two additional identical decoupling gratings (including three air grooves) were designed on both sides with a distance of 8 μm away from the nano-slit. In order to learn the propagation loss of SPP wave, we calculated the imaginary part of the effective index of SPP and the corresponding propagation length of the Au/PVA/Air configuration (see the Supplementary Fig. S2). The average imaginary part of SPP is about 0.007, and the average propagation length of SPP in the Au/PVA/Air configuration is about 10 μm from 780 nm to 980 nm, so the 8-μm distance of the decoupling grating to the nano-slit is enough for the CCD detection in the experiment. The period of the grating is 520 nm, the grooves width and depth are 260 nm and 250 nm, respectively. As for the real sample, the PVA powder with an average molecular weight of 30,000 was dissolved in de-ionized water with a weight ratio of 1:31. The spin coating method was adopted to fabricate the PVA layer on the surface of gold films. The real thickness was approximate to 150 nm for PVA layer and 300 nm for gold film. A focused-ion-beam (FIB) etching system (Model Helios NanoLab 600, FEI Company, USA) was employed to prepare the patterns of the nano-slit and the grooves on the Au/PVA platform directly according to the designed structure parameters. The spot current of the ion beam was only 7.7 PA in order to improve the etching quality. The length of the fabricated sample in the Z direction was 20 μm, which is much larger than the incident wavelength to make sure the two-dimensional simulation model is completely exact. The 20-μm dimension is used to eliminate the influences of the roughness of the gold film and the etching error, since the calculation is based on the 2D design. In fact, the dimension in the Z-direction can be shortened to much less than 20 μm by use of the 2D design^6^,^32^. When the dimension is shortened to the order of the wavelength, the device still possesses excellent properties according to our calculation, which is shown in Supplementary Fig. S3. The scanning-electron-microscopy (SEM) image of the SPP demultiplexer sample is shown in Fig. 2(b). We calculated the leftward power flow of the left side the left MCC in the PVA layer and the rightward power flow of the right side of the right MCC for different incident wavelengths, which is shown in Fig. 2(c). It shows that the left intensity is more than the right intensity from 750 nm to 880 nm; while the right intensity is more than the left intensity from 900 nm to 1050 nm. To demonstrate the properties of the wavelength demultiplexer more directly, we take the incident wavelength of 840 nm and 920 nm for examples, the calculated the power flow and the field distribution are shown in Fig. 2(d,e). From Fig. 2(d), we can see there is strong power flow distribution of the SPP mode in the PVA layer instead of that in the air, and the power flow only propagates in the left direction, while the rightward power is near to zero. The field distribution of Hz component for SPP in Fig. 2(e) demonstrates that SPPs are confined at the interface of the gold film and PVA layer, so very tight field is formed in our configuration. The SPPs propagate leftward for the incident wavelength of 840 nm, and propagate rightward for the incident wavelength of 920 nm. Figure 2(e) also confirms that it is the second order of the SPP mode that exists in the MCC, i.e. there exists two periods of the effective wavelength of SPP, which is in agreement with the derivation result from formula (1). The PVA layer ensures an ultracompact field, so the nano-cavity on the Au/PVA/Air configuration can generate efficient modulation on SPPs compared with the Au/Air configuration^19^. It should be pointed out that the transmission efficiency of the MCC is near to that without the multicomponent cavity at some wavelength, as shown in Supplementary Fig. S1, in other words, the existence of the MCC has little influence on the transmission efficiency of SPP compared with the structure only with the nano-slit. It can be seen that for the wavelength from 780 nm to 890 nm, the leftward power flow is a little higher than the rightward power flow, and the transmission efficiency of the structure with the multicomponent cavity is approximately 20% higher than that of the structure without the multicomponent cavity (i.e. the reference); for the wavelength from 890 nm to 980 nm, the rightward power flow is a little lower than the leftward power flow, and the transmission efficiency of the structure with the multicomponent cavity is approximately 15% lower than that of the structure without the multicomponent cavity (i.e. the reference), since the incident power flow is constant, when the leftward power flow is increased, the rightward power flow is decreased.

**Figure 3:**
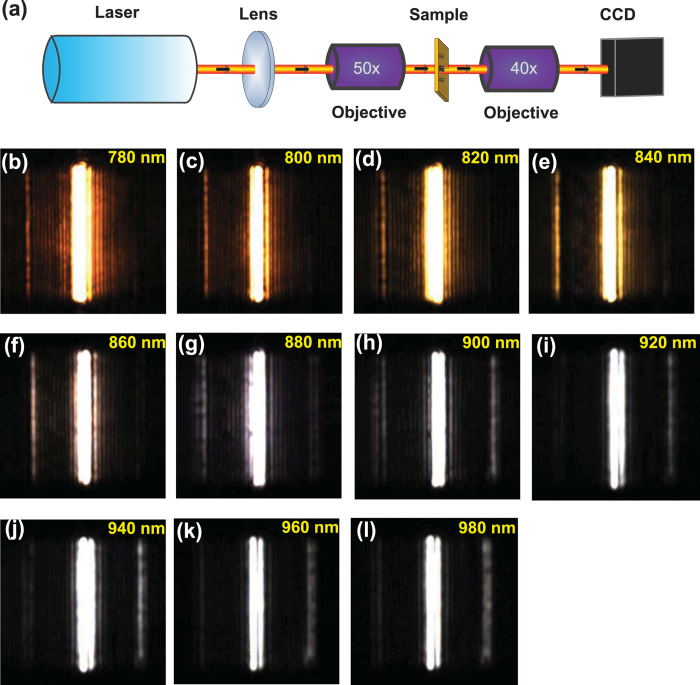
Experimental demonstration of the wavelength demultiplexer. (**a**) Schematic diagram for the experimental setup. The sample is illuminated by the normal incident focused laser from the backside, and the scattered intensity of the surface of the sample is collected by the CCD. The captured CCD images [from (**b**–**l**)] for the measured intensity of the sample surface for different incident wavelengths from 780 nm to 980 nm with 20 nm per step.

**Figure 4:**
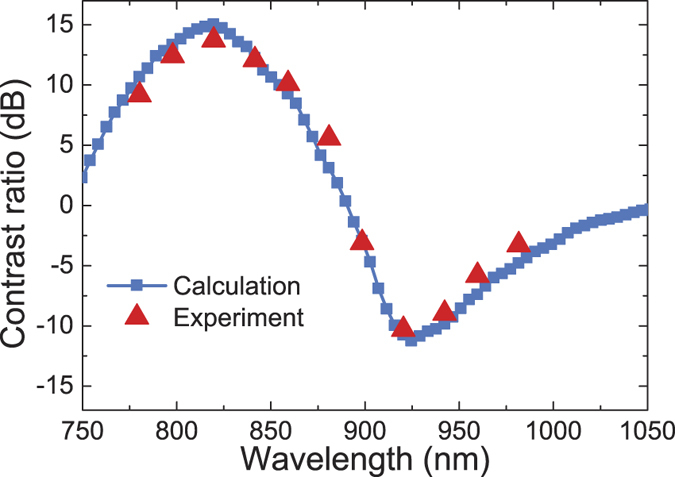
Contrast ratio for different wavelength. Calculated (blue squre) and experimental (red triangle) contrast ratio for the left intensity to the right intensity for different incident wavlength, respectively.

In our experiment, an optical micro-spectroscopy measurement system was adopted to measure the functions of the SPP wavelength demultiplexer, which is shown in Fig. 3(a). The nano-slit was normally illuminated from the back side using a p-polarized continuous wave Ti:sapphire laser beam with different wavelengths. The optical-thick gold film can prohibit the direct transmission of the incident laser beam. The incident laser beam was focused into a spot with a radius of about 50 μm, ensuring uniform illumination of the entire nano-slit. The line width of the laser spectrum curve was about 1.5 nm, which ensures that only needed quasi-monochromatic SPP mode can be excited by the SPP wavelength demultiplexer^18^. The SPP mode was scattered by two decoupling gratings in the output ports. The scattered light was collected by a long working distance objective (Olympus 40×, NA 0.65) and then imaged onto a CCD (Microview, MVC1300F). Figure 3(b–l) show the captured CCD images for different incident wavelengths from 780 nm to 980 nm by 20 nm per step. The bright and dark contrast at the left decoupling grating and the right decoupling grating is very clear for different incident wavelengths. It is obvious that the intensity at the left decoupling gratings is larger than that at the right decoupling gratings from 780 nm to 880 nm; while the intensity at the right decoupling gratings is larger than that at the left decoupling gratings from 780 nm to 880 nm. The intensity values at the left decoupling gratings and the right decoupling gratings can be obtained from the CCD images. The contrast ratio can be obtained from 10·log(I_L_/I_R_), where I_L_ and I_R_ are the scattered light intensity from the left and right decoupling gratings, respectively, which are extracted from the CCD images in Fig. 3(b–l). The method through measuring scattered light intensity to demonstrate the nanoscale device property is widely used in a variety of plasmonic devices^6^,^8^,^11^,^13^,^18^,^19^,^20^,^21^. The contrast ratio of the scattered light intensity is equal to that of the transmitted intensity due to the identical decoupling gratings at the rightside and leftside of the structure. The weak fine fringes between the decoupling gratings and the nano-slit come from the transmitted SPPs from the MCC and the reflected SPPs from the decoupling gratings. The intensity along the decoupling grating in the Z direction is not uniform, which is caused by the roughness of the film (see Supplementary Fig. S4) and the imperfect etching process (see Supplementary Fig. S5). The roughness of the gold film and the PVA films is about 10 nm and 4 nm, respectively. The etching accuracy of the FIB etching system is 10 nm. The calculated and experimental contrast ratio is shown in Fig. 4 for different incident laser wavelengths, where the experimental results are in agreement with the calculated results using finite-element method. For 840-nm incidence, the intensity is 668075 a.u. at the left decoupling gratings and 25021 a.u. at the right decoupling gratings (see Fig. 3d), so the experimental contrast ratio is 13.7 dB; while for 920-nm incidence, the intensity is 686100 a.u. at the right decoupling gratings and 64121 a.u. at the right decoupling gratings (see Fig. 3h), so the contrast ratio is −10.3 dB. The experimental average contrast ratio reaches 10.5 dB for the short wavelength range from 780 nm to 880 nm, and −7.1 dB for the long wavelength range from 900 nm to 980 nm, which is among the highest contrast ratio compared with the previous reported values^1^,^2^,^5^,^6^,^7^,^8^,^9^,^10^,^13^. The operating bandwidth is enlarged almost one magnitude compared with previous experimental reported values^5^,^6^,^11^. The broadband is mainly due to the broad linewidth of the MCC. The contrast ratio of the wavelength demultiplexer based on Au/PVA/Air configuration is higher than that of the structure based on the Au/Air configuration^5^,^6^,^8^. However, the coupling efficiency of the light from free-space to the chip is worth improving. For example, some more efficient structure of the unidirectional SPP launcher^19^, focused SPP circle gratings^33^,^34^, and even tapered plasmonic nanoantennas can be adopted at the input port^35^. In addition, the direct jointing by use of on-chip ultrasmall laser is also an alternative way, i.e. the on-chip ultrasmall laser is directly used as the input port instead of the coupling of the free-space laser to on-chip device.

In summary, we experimentally realized a broadband ultracompact plasmonic wavelength demultiplexer by adopting two asymmetric MCCs on a slab configuration of Au/PVA/Air. The operation wavelength range is from 780 nm to 980 nm, the feature size is only 2.3 μm for the whole device, and the contrast intensity ratio is 13.7 dB for the short wavelength, and −10.3 dB for the long wavelength. Moreover, the splitting wavelengths and their separation can be easily adjusted by varying the structural parameters for the proposed MCC configuration. The MCC configuration is very easy for fabrication. The planar configuration of the device is much suitable for the practical on-chip integration applications. This broadband plasmonic wavelength demultiplexer may have ample scope for its abilities in optical communication rely on structures that collect and sort photons by wavelength.

## Methods

### Numerical simulation

Numerical simulations were performed by use of the commercial finite element (FEM) solver of COMSOL Multiphysics. The refractive index of the dielectric film was 1.50^18^, and the permittivity of the gold was calculated as a function of the wavelength using interpolation and was taken from ref. 36. Here, the two-dimensional frequency domain module and mode analysis module were used.

### Sample fabrication

The gold film was fabricated using a laser molecular beam epitaxy (LMBE) growth system (Model LMBE 450, SKY Company, China). The beam (wavelength 248 nm, a pulse repetition rate 3 Hz) output from an excimer laser system (Model COMPexPro 205, Coherent Company, USA) was used as the light source. The beam is focused onto a gold target mounted on a rotating holder, 15 mm away from the silicon dioxide substrate. A typical energy density of the excitation laser is about 500 mJ/cm^2^. The growth rate measured by a film thickness/rate monitor is about 0.01 nm/pulse. PVA powder with an average molecular weight of 30,000 (J&K company, China) is dissolved in de-ionized water with a weight ratio of 1:31. The spin coating method is used to fabricate the PVA layer on the surface of the gold films. A FIB etching system (Model Helios NanoLab 600, FEI Company, USA) is employed to prepare the patterns of the nanoslit and the 1D plasmonic crystal. The spot current of the ion beam was only 7.7 pA to improve the etching quality.

### Micro-spectroscopy measurement setup

A micro-spectroscopy measurement system is used to measure the intensity of the sample surface. The nanoslits are normally illuminated from the back side using a p-polarized CW Ti:sapphire laser (Model Mira 900F, Coherent Company, USA) with different wavelengths. The optical-thick gold film can prohibit the direct transmission of the incident laser beam. The line width of the laser spectrum curve is about 1.5 nm which ensures that only the specified quasi-monochromatic SPP modes can be excited by the nanoslit. The SPP mode is scattered using decoupling grating in the output port. The scattered light is collected by a long working distance objective (Mitutoyo 20, NA = 0.58) and then imaged onto a charge coupled device (CCD).

## Additional Information

**How to cite this article**: Lu, C. *et al.* Integrated ultracompact and broadband wavelength demultiplexer based on multi-component nano-cavities. *Sci. Rep.* **6**, 27428; doi: 10.1038/srep27428 (2016).

## Supplementary Material

Supplementary Information
