# Supplementary material for: Integrated ultracompact and broadband wavelength demultiplexer based on multi-component nano-cavities
Source: Sci Rep. 2016 Jun 6;6:27428. doi: 10.1038/srep27428 (PMC4893661; doi:10.1038/srep27428)
Supplement: Supplementary Information [file srep27428-s1.doc]

**Supplementary Information**

Subject areas: nanophotonics and plasmonics, Subwavelength OPTICS, INTEGRATED OPTICS, APPLIED PHYSICS

Correspondence and requests for materials should be addressed to C. C. Lu. (lucuicui@qxslab.cn)

**Integrated ultracompact and broadband wavelength demultiplexer based on multi-component nano-cavities**

Cuicui Lu^1^, Yong-Chun Liu^1^, Xiaoyong Hu^2,3^, Hong Yang^2^ & Qihuang Gong^2,3^

^1^ Qian Xuesen Laboratory of Space Technology, China Academy of Space Technology, Beijing 100094, People’s Republic of China

^2^ State Key Laboratory for Mesoscopic Physics & Department of Physics, Peking University, Beijing 100871, People’s Republic of China

^3^ Collaborative Innovation Center of Quantum Matter, Beijing 100871, People’s Republic of China

**The supplementary information includes:**

I. Power flow of the transmitted light with/without the multicomponent cavity

II. Loss of SPP in the Au/PVA/Air configuration

III. Power flow distributions of the 3D model

IV. AFM images for the film surface

V. SEM image for the different etching depth of the grooves

**I. Power flow of the transmitted light with/without the multicomponent cavity**

**Figure S1. Calculated power flow of the transmitted light by use of the finite element method. The blue square line denotes the power flow of the leftside of the multicomponent cavity; the red triangle line denotes the power flow of the rightside of the multicomponent cavity; and the dark circle line denotes the power flow of the rightside (or leftside) of the nanoslit without the multicomponent cavity, where is the reference power flow.**

**II. Loss of SPP in the Au/PVA/Air configuration**

**Figure S2. Calculated the imaginary part of SPP effective index and the corresponding propagation length by use of the finite element method. The red triangle line denotes the imaginary part of SPP effective index of the Au/PVA/Air configuration; the blue square line denotes the corresponding propagation length of the Au/PVA/Air configuration.**

**III. Power flow distributions of the 3D model**


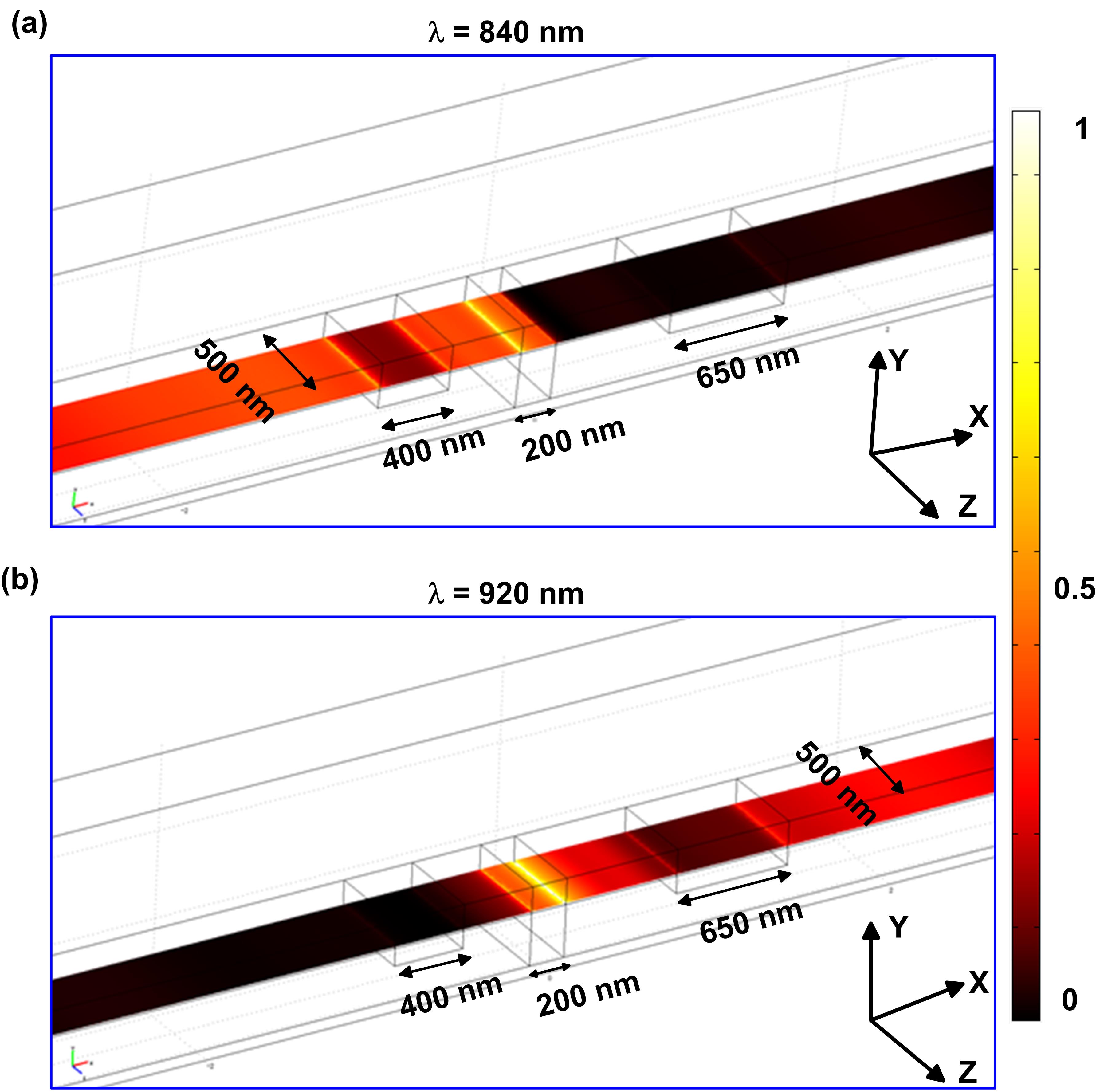


**Figure S3. Calculated the power flow distribtutions in the ZX plane of 10-nm above the the surface of the gold film for different incident wavelength. (a) When the incident wavelength is 840 nm, the SPPs propagate in the leftward direction; (b) When the incident wavelength is 920 nm, the SPPs propagate in the rightward direction.**

**IV. AFM images for the film surface**


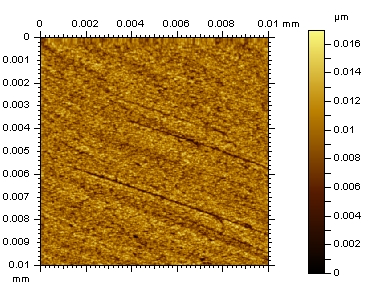


**(a)**


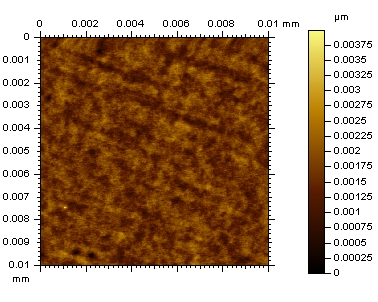


**(b)**

**Figure S4. Atomic force microscope (AFM) images the 300nm-thick gold film (a) and the 150nm-thick PVA on gold film (b).**

**V. SEM image for the different etching depth of the grooves**


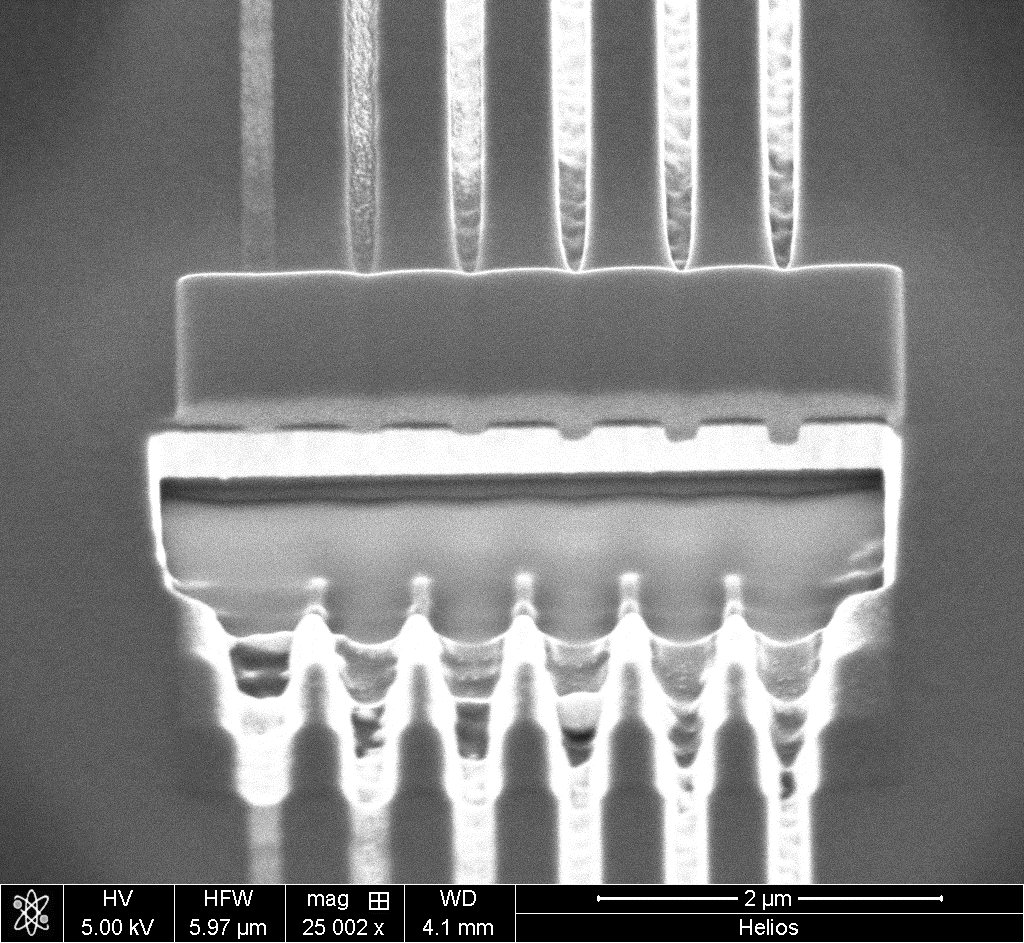


**Au**

**Pt**

**SiO2**

**PVA**

**Figure S5. SEM image for the different etching depth of the grooves.**
